# Antioxidant activity of selenium-enriched *Chrysomyia megacephala* (Fabricius) larvae powder and its impact on intestinal microflora in D-galactose induced aging mice

**DOI:** 10.1186/s12906-020-03058-4

**Published:** 2020-08-27

**Authors:** Dandan Xie, Liqin Jiang, Yao Lin, Zhenwei Liu

**Affiliations:** 1 College of Pharmaceutical Science, Zhejiang Chinese Medical University, 548 Binwen Road, Hangzhou, 310053 China; 2 Beijing Ershang Biological Technology Co., Ltd, 234 Shiliuzhuang West Road, Beijing, 100053 China

**Keywords:** Selenium-enriched *Chrysomyia Megacephala* (Fabricius) larvae powder (SCML), Antioxidant activity, Nrf2, Intestinal microbiota, In vivo

## Abstract

**Background:**

The purpose of this study was to assess the antioxidative activity of selenium-enriched *Chrysomyia Megacephala* (Fabricius) (*C. megacephala*) larvae powder (SCML) and its impact on the diversity and structure of intestinal microflora in a mouse model of D-galactose (D-gal)-induced oxidative damage.

**Methods:**

Sixty male ICR mice were equally randomized to a normal control (NC) group, a model group, a positive group, a low-dose SCML (L-SCML) group, a mid-dose SCML (M-SCML) group, and a high-dose SCML (H-SCML) group. Animals in NC and model groups received water, animals in the positive group received 40 mg/Kg vitamin E (VE), and those in the three SCML groups received SCML which include 300, 1000 and 3000 μg/Kg selenium (Se) respectively. An oxidative damage model induced by subcutaneous injection of D-gal for 6 weeks via the neck was established. Serum oxidative stress levels and tissue appearance were evaluated. Tissues oxidative stress levels were detected by commercially available kit. Nuclear erythroid 2-related factor (Nrf2) and gut microbiota were determined by western blot and high throughput sequencing 16S rRNA gene respectively.

**Results:**

An oxidative damage model was established successfully as represented by a significant elevation of malondialdehyde (MDA) and protein carbonylation, and inhibition of the antioxidants including superoxide dismutase (SOD), glutathione peroxidase (GSH-Px), total antioxidant capacity (T-AOC) and glutathione (GSH). It was found that oxidative damage and histological alterations were attenuated, the expression of Kelch-like ECH-associated protein (Keap1) was decreased, and the expression of Nrf2 and hemeoxygenase-1 (HO-1) was increased after SCML treatment. In addition, significant changes were observed in the gut microbiota, including *Proteobacteria* and the ratio of *Bacteroidetes* to *Firmicutes* at the phylum level, as well as *Helicobacter*, *Clostridium* and *Lactobacillus* at the genus level.

**Conclusion:**

SCML exerted an antioxidative effect in vivo, probably by increasing the antioxidant activity and reducing the production of oxidation products via the Nrf2 signaling pathway. SCML could also redress the intestinal flora imbalance induced by oxidative stress. All these findings suggest that SCML could serve as a functional food and natural drug additive to protect the human body against oxidative damage.

## Background

Aging is a natural process that involves the gradual loss of physiological functions, causing enhanced morbidity and mortality due to various diseases. This process is closely related to oxidative stress [1–3]. One prevalent theory to explain aging is the theory of the oxygen free radical [4]. This theory posits that the macromolecules (such as nucleic acids, lipids, sugars, and proteins) that make up cells and tissues are subjected to oxidative stress induced by superoxide and other free radicals. These macromolecules then undergo different degrees of oxidation, which initiates oxidative damages and ultimately leads to organ function impairment and aging [5, 6]. Changes in the level of oxidative stress affect the microbial environment in the intestine and lead to intestinal flora disorder [7]. Disordered intestinal flora may affect the antioxidant activity and lipid metabolism [8]. Hence, it may be possible to inhibit oxidative stress by regulating the composition and structure of the gut flora. To prevent oxidative stress-associated cellular damage, it is therefore important to keep prooxidant-antioxidant balance by supplementation or induction of cellular antioxidants. A high dose of d-galactose is converted to aldose and hydrogen peroxide by d-galactose oxidase. The products then generate reactive oxygen species through oxidative metabolism and glycosylation, leading to oxidative stress. The accumulation of oxidation products further exacerbates the oxidative damage to tissues and cells, which then accelerates the aging process [9]. Therefore, d-galactose overload has been used to establish animal models used to conduct aging related metabolic dysfunction and oxidative stress [10, 11].

Selenium (Se) is an essential trace element for human body and other animals. The role of Se is reported to be closely associated with antioxidant activity, immune response, and chemoprevention [12–15]. Se is mainly present in the active site of enzymes in the form of selenocysteine. Multiple Se-containing proteins such as GSH-Px and thioredoxin reductase play important roles in preventing oxidative injury [16]. Therefore, the importance of Se supplementation in boosting up the internal antioxidative defense has been highlighted in recent years. Studies have shown that organic Se supplements can improve tissue Se deposition, antioxidant level, and gene expression, whereas Se deficiency may result in cardiac, muscular, osseous and immune disturbances [17, 18]. Therefore, the health-related benefits of Se including the type of selenium supplements and optimal dosage remain to be explored.

The importance of Se has inspired researchers to use bioenrichment to prepare high Se compounds [19, 20]. *C. megacephala* larvae is a traditional Chinese medicine with a wide range of pharmacological actions, including antioxidant, antibacterial, and anti-inflammatory activities, which has been widely applied in agriculture and medicine [21–23]. Se-enriched *C. megacephala* larvae (SCML) is generated from *C. megacephala* larvae by biological transformation and enrichment of Se. Our previous work showed that SCML was an effective organic Se source with low toxicity and high Se content [24]. Yet, no study has reported the antioxidant activity of SCML in vivo and its impact on the gut microbiota, which is susceptible to undergo alterations under oxidative stress.

The objective of the present study was to evaluate the antioxidant activity of SCML in vivo, explore the underlying mechanism, as well as evaluate its impact on the gut microbial diversity and structure, hoping that the results could provide a scientific basis for a comprehensive utilization of SCML.

## Methods

### Materials and chemicals

SCML was provided by Beijing Ershang Biological Technology Co., Ltd. (Beijing, China). Vitamin E was purchased from Archer Daniels Midland (Dictor, USA). D-galactose (D-gal) of ≥99% purity was purchased from Aladdin Industrial Corporation (Shanghai, China). GSH-Px, SOD, T-AOC, GSH, MDA and protein carbonyl assay kits were purchased from Nanjin Jiancheng Bioengineering Institute (Nanjin, China). RNA trizol reagent and FastStart Universal SYBR Green Master (Rox) were purchased from Servicebio (Wuhan, China). The primers for Nrf2, SOD1, GSH-Px and GAPDH were synthesized and purified by Wuhan Servicebio Technology Co., LTD (Wuhan, China). The kits for Revert Aid First Strand cDNA synthesis and HyPure™Molecular Biology Grade Water were purchased from Thermo (Waltham, USA) and HyClone (Logan, USA) respectively. Keap1, Nrf2 and HO-1 polyclonal antibodies were obtained from Proteintech (Chicago, USA). RIPA, β-Actin, bicinchoninic acid (BCA) assay kit, Western Lightening™ Plus-ECL Enhanced chemiluminescence substrate assay kit and the secondary goat anti-mouse horseradish peroxides (HRP) were from Servicebio (Wuhan, China). All other chemicals and reagents used in the study were of analytical grade. Water used in the experiments was ultrapure.

### Determination of the compositions of SCML

Compositions of SCML including protein, crude fat and moisture content were analyzed according to method GB5009–2016 of China National Food Safety Standard. Se content was detected by Inductively Coupled Plasma (ICP) according to Vu et al with minor modifications [25]. The results are shown in Table 1.

**Table 1:** Compositions of SCML

	Protein (g/100 g)	Crude Fat (g/100 g)	Moisture (g/100 g)	Se (μg/g)
Content	54.6	15.0	6.6	9000

SCML: Selenium-enriched *C. megacephala* larvae powder

### Animal experiments

Sixty ICR male mice aged 5 weeks and weighing 20 ± 2 g were purchased from Sino-British SIPPR/BK Lab. Animal Ltd. (Approval No. SCXK (HU) 2013–0016). The animal experiments were performed in accordance with the guidelines of the Laboratory Animal Center of Zhejiang Chinese Medical University (Permit No. SYSK (ZHE): 2018–0012). All the experimental procedures were strictly conducted according to the international standards and national legislation on animal care and use. The mice were kept under controlled light conditions (12 h light-dark cycle) with free access to food and water, normal light circadian rhythm and 7-day adaptive feeding in a quiet environment.

After one-week acclimatization, 60 mice were equally randomized to six groups: 1) normal control (NC) group, 2) model group, 3) positive group receiving 40 mg/Kg·d vitamin E (VE group), 4) low-dose SCML (L-SCML) group receiving SCML (300 μg/Kg·d Se), 5) mid-dose SCML (M-SCML) group receiving SCML (1000 μg/Kg·d Se), and 6) high-dose SCML (H-SCML) group receiving SCML (3000 μg/Kg·d Se). Except for the mice in NC group, animals in the other five groups were given subcutaneous injection of 200 mg/Kg·d D-gal for 6 weeks into the neck to prepare oxidative stress model. Animals in NC and model groups received water, and animals in the other groups as previously described received VE or SCML by intragastric gavage for 6 weeks. The experiments were conducted at 9:00–12:00. A certain amount of SCML and gellan gum were weighed precisely and dissolved in purified water, heated slightly to a suspension. There were three different concentrations: 30, 100, and 300 μg/mL Se. Meanwhile VE was dissolved in purified water containing gellan gum, which became a suspension (4.0 mg/mL). D-gal was dissolved in 0.9% physiological saline (20.0 mg/mL).

The mice were weighed throughout the experiment. The appearance, appetite, mental condition and behavioral activity of the mice during the experiment were also observed and recorded. Stool samples were collected at 5 weeks after treatment. Blood samples were obtained from the retrobulbar venous plexus at 6 weeks after treatment. The mice were sacrificed by cervical dislocation and the liver, kidney, heart, brain, and caecum were stripped. The dissected organs were divided two parts (one for histological analysis and the other for biochemistry analysis). Samples for analysis were thawed on ice, homogenized with 5-10 mL cold buffer (50 mM potassium phosphate with 1 mM EDTA, pH 7) per gram of tissue, and centrifuged at 10,000×g for 15 min at 4 °C. The supernatants were collected for analysis.

### Analysis of serum oxidative stress indexes

Serum oxidative stress indexes GSH-Px, SOD and MDA were determined by using the respective commercial kits according to the manufacturer’s instructions.

### Analysis of tissue oxidative stress indexes

The oxidative stress indexes were determined by measuring GSH-Px, SOD, T-AOC, GSH, MDA and protein carbonylation of the tissue homogenate supernatant using the commercial kits according to the manufacturer’s instructions.

### Histological analysis

For histological analysis, the animal tissues were fixed in 4% paraformaldehyde for 24 h, dehydrated in alcohol, paraffin embedded, sliced into 4 μm thick sections, stained with hematoxylin-eosin (HE), and finally photographed under a microscope (40× objective lens).

### RNA extraction and real-time quantitative PCR experiments

Total RNA was extracted from the liver and kidney tissues using Trizol reagent. RNA was reverse transcribed into cDNA using RevertAid First Strand cDNA Synthesis Kit. Real-time quantitative PCR (qRT-PCR) was performed using FastStart Universal SYBR Green Master (Rox) and the ABI7900Faxt Sequence Detection system. The thermal cycle condition was 1 cycle at 95 °C for 10 min, followed by 40 cycles of amplification at 95 °C for 15 s, and then 60 °C for 30s. And the dissolution curve started from 60 °C, then ascending to 95 °C at 0.3 °C/15 s. All samples were run in triplicate in each experiment. Values were normalized to that for GAPDH. The sequences of the primers used are shown in Table 2. The results were calculated by using the 2^-ΔΔCT^ method.

**Table 2:** Primers for real-time PCR analyses

Gene	Accession No.	Primer Sequences (5`-3`)	Product Size/bp
Nrf2	NM_010902.3	CTGGCTGATACTACCGCTGTTC	208 bp
		AGGTGGGATTTGAGTCTAAGGAG	
SOD1	NM_011434.1	ATGTGACTGCTGGAAAGGACG	200 bp
		CGCAATCCCAATCACTCCAC	
GSH-Px	NM_008160.6	CCAGGAGAATGGCAAGAATGA	137 bp
		GGAAGGTAAAGAGCGGGTGA	
GAPDH	NM_008084.2	CCTCGTCCCGTAGACAAAATG	133 bp
		TGAGGTCAATGAAGGGGTCGT	

### Western blot analysis

Total protein and nuclear protein were extracted from 100 mg liver and kidney tissues using Radio-Immunoprecipitation Assay (RIPA) lysis solution and a nuclear/cytoplasm protein extraction kit. The concentrations of protein lysates were quantified using a BCA protein kit. Samples containing an equal amount of protein (20 μg) were mixed with the loading buffer containing 5% 2-mercaptoethanol, heated for 10 min at 99 °C, and loaded onto a 10% SDS-PAGE gel. The proteins from the electrophoresing gel were then transferred onto polyvinylidene difluoride membranes, which then blocked with 5% milk and 0.1% Tween 20 in Tris-buffered saline, and incubated overnight at 4 °C with anti-Keap1 (1:1000), anti-Nrf2 (1:1000), anti-HO-1 (1:1000) and β-actin (1:3000). Then, the appropriate horseradish peroxide-conjugated secondary antibody was added to the membranes at room temperature. Finally, the proteins were detected with chemiluminescent substrate. Gray semi-quantitative analysis was performed by Image J. The protein bands were quantified using densitometry. Values are expressed as the fold change with respect to beta-actin.

### Intestinal microbiota analysis

The stool samples were sent to BGI Co., Ltd. (Wuhan, China) for sequencing of the 16S rRNA gene. Total genomic DNA of the gut microbiome was extracted, and the V3-V4 region of the 16S rRNA gene from the sample was subjected to PCR amplification. After normalization of the genome DNA to 30 ng per PCR reaction, V3-V4 dual-index fusion PCR primer cocktail and PCR master mix were added, and then a PCR was performed. The PCR products were purified with Agencourt AMPure XP beads to remove the unspecific products. Paired-end sequencing was performed on the Illumina Hiseq platform and the obtained data were subjected to bioinformatics analysis.

To obtain clean reads, the clean paired-end reads with overlap were merged to tags using FLASH (fast length adjustment of short reads, v1.2.11). Then, the tags were clustered to operational taxonomic units (OTUs) at 97% sequence similarity by scripts of software USEARCH (v7.0.1090). The RDP classifier (v2.2) was used to compare OTUs with the database to comment on the OTUs species. Finally, intestinal microbial diversity and structure were analyzed based on OTUs and taxonomic ranks using software R (v3.1.1).

### Statistical analysis

All data are expressed as the means ± SD or means ± SE and analyzed using Statistical Analysis Software (SPSS 20.0). The experimental values were analyzed by one-way ANOVA followed by the Duncan’s multiple-range tests, and *P*-value < 0.05 were considered to be statistically significant.

## Results

### Effects of SCML on daily behavior and weight gain in mice

**Fig. 1:**
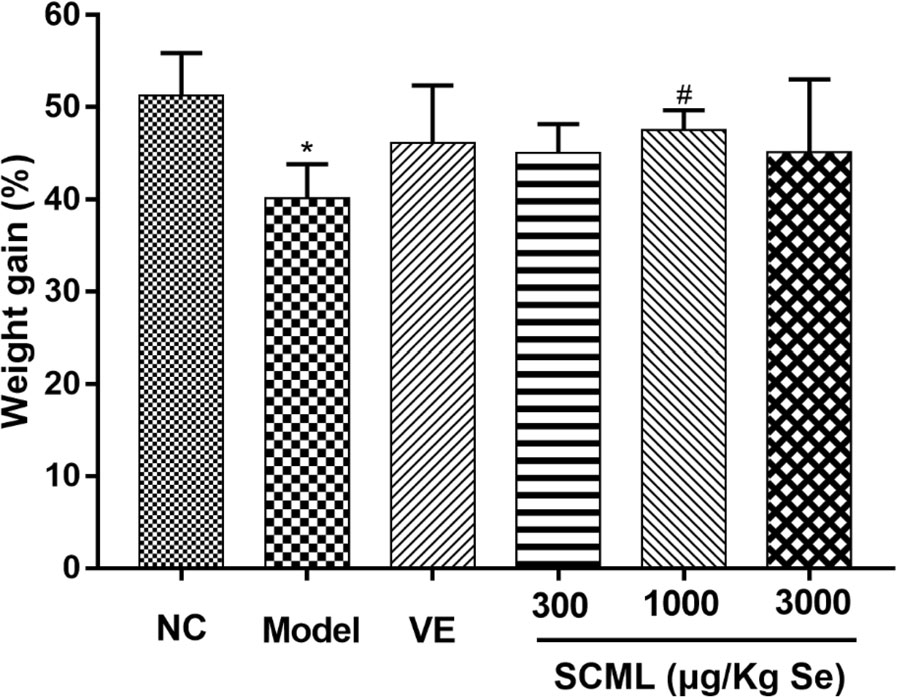
Percentage of weight gain in mice at 6 weeks. Values represent means ± SD (*n* = 10) and evaluated by one-way ANOVA followed by the Duncan’s multiple-range tests. Compared with NC, ^*^P < 0.05; Compared with Model, ^#^*P* < 0.05. SCML: Selenium-enriched *C. megacephala* larvae powder

Usual performance of the mice was observed and recorded, and no abnormal phenomenon found during the experiment, including anti-feeding and vomiting. Symptoms such as slow movement and listlessness were obviously observed in model group, indicating that the oxidative stress model induced by subcutaneous injection of D-gal was successfully established. However, the above symptoms receded in varying degrees in VE and SCML groups. The weight gain of the mice is exhibited in Fig. 1. Compared with NC group, body weight of the mice in model group significantly increased slowly (*P* < 0.05), and increased steadily in drug treatment groups. M-SCML (1000 μg/Kg Se) group showed a significant difference compared to the model group (*P* < 0.05).

### Effects of SCML on serum oxidative stress indexes in mice

**Fig. 2:**
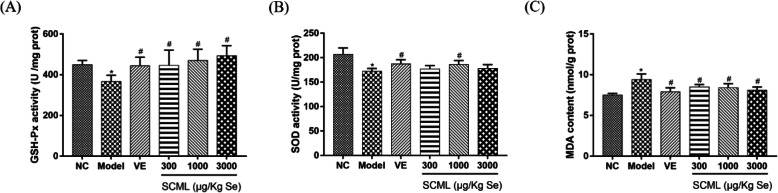
Oxidative stress level indexes of the mice serum. **a** GSH-Px activity in the mice serum, **b** SOD activity in the mice serum, **c** MDA content in the mice serum. Values represent means ± SD from three independent replicates (*n* = 10) and evaluated by one-way ANOVA followed by the Duncan’s multiple-range tests. Compared with NC, ^*^P < 0.05; Compared with Model, ^#^*P* < 0.05. SCML: Selenium-enriched *C. megacephala* larvae powder

As shown in Fig. 2, the serum antioxidative enzyme activities in model group were decreased significantly and the MDA content was increased significantly compared to NC group (*P* < 0.05). As shown in Fig. 2a, the GSH-Px activities in animals treated with SCML or VE were increased significantly (*P* < 0.05). As shown in Fig. 2b, the activities of SOD in M-SCML (1000 μg/Kg Se) and VE groups were significantly increased compared to model group (*P* < 0.05). As shown in Fig. 2c, the MDA levels in animals treated with SCML or VE were decreased significantly (*P* < 0.05).

### Effects of SCML on tissue oxidative stress indexes in mice

**Fig. 3:**
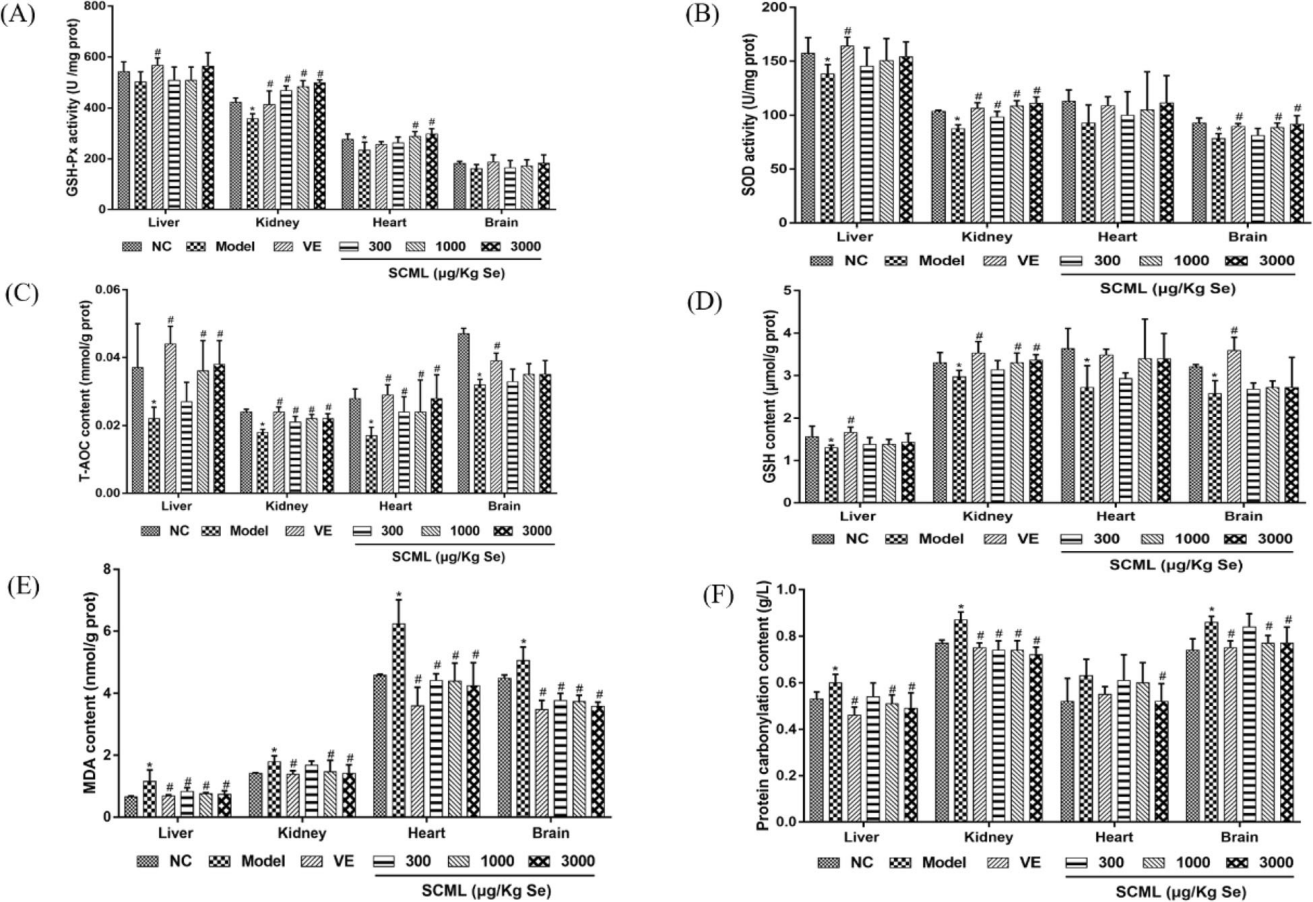
Oxidative stress indexes of the mice tissue. **a** GSH-Px activity in the mice tissue, **b** SOD activity in the mice tissue, **c** T-AOC content in the mice tissue, **d** GSH content in the mice tissue, **e** MDA content in the mice tissue, **f** protein carbonylation content in the mice tissue. Values represent means ± SD from three independent replicates (*n* = 10) and evaluated by one-way ANOVA followed by the Duncan’s multiple-range tests. Compared with NC, ^*^P < 0.05; Compared with Model, ^#^*P* < 0.05. SCML: Selenium-enriched *C. megacephala* larvae powder

As illustrated in Fig. 3, after 6-week subcutaneous injection of D-gal, the activity of the antioxidative enzymes and the content of the antioxidants in different mice tissues were decreased significantly compared to NC group (*P* < 0.05), except for SOD in the heart as well as GSH-Px in the liver and brain. After administration of VE or SCML, the activity of GSH-Px and SOD, as well as the content of T-AOC and GSH were increased gradually. As shown in Fig. 3a, the activity of GSH-Px in the kidney and heart was increased significantly, compared to model group (*P* < 0.05), except for the heart in VE group and L-SCML (300 μg/Kg Se) group. The activity of GSH-Px in the liver and brain remained unchanged significantly, compared to model group, except for VE group in the liver. As shown in Fig. 3b, the activity of SOD in the kidney and brain was increased significantly, compared to model group (*P* < 0.05), except for the brain in L-SCML (300 μg/Kg Se) group. However, the activity of SOD in the liver and heart remained unchanged significantly, compared to model group, except for the liver in VE group. As seen in Fig. 3c, the content of T-AOC in the liver, kidney and heart was increased significantly compared to the model group (*P* < 0.05), except for the liver in L-SCML (300 μg/Kg Se) group. The content of T-AOC in the brain was not significantly altered compared to model group, except for VE group. As shown in Fig. 3d, the content of GSH in liver, kidney and brain of VE group, and in the kidney of H-SCML (3000 μg/Kg Se) and M-SCML (1000 μg/Kg Se) groups was increased significantly compared to model group (*P* < 0.05).

As shown in Fig. 3e, the MDA level in model group was increased significantly compared to NC group (*P* < 0.05), and decreased significantly after VE or SCML treatment compared to model group (*P* < 0.05), except for L-SCML (300 μg/Kg Se) group in the kidney. In Fig. 3f, the protein carbonylation level in the liver, kidney and brain of model group was increased significantly compared to NC group (*P* < 0.05). However, the level decreased significantly in the mice treated with SCML or VE, except for in the liver and brain of L-SCML (300 μg/Kg Se) group compared to model group (*P* < 0.05). And compared with model group, H-SCML (3000 μg/Kg Se) group in heart also significantly decreased in protein carbonylation level.

### Effects of SCML on histopathological changes in mice

**Fig. 4:**
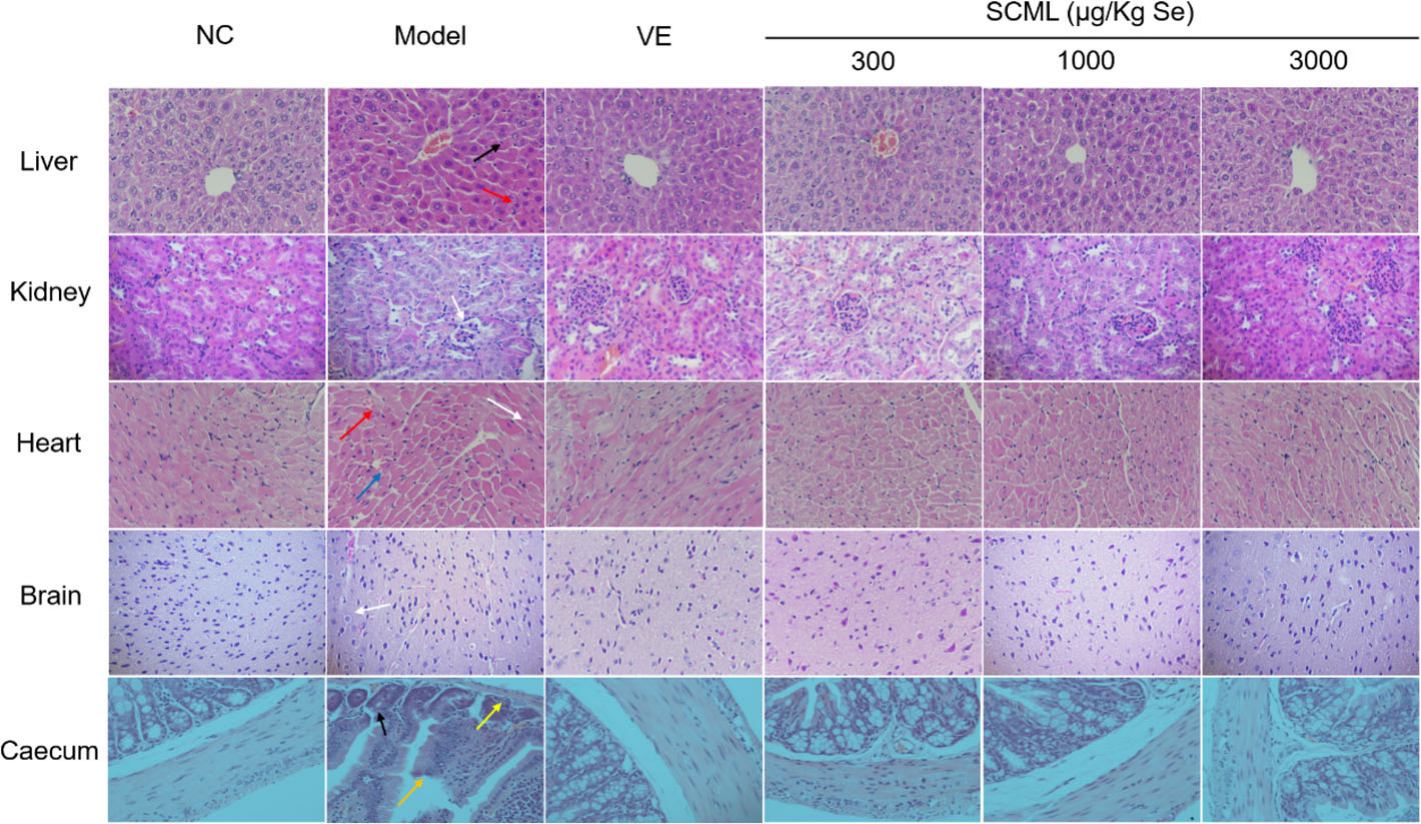
Optical micrographs of mice tissue sections (HE staining 400×). Black arrow: derangement of hepatic cord cells, Red arrow: infiltration of inflammatory cells, White arrow: pyknosis, Blue arrow: cavitation and deformation, Orange arrow: atrophy and breakage of the villus, Yellow arrow: thinning of the intestinal wall. Scale bar:50 μm. SCML: Selenium-enriched *C. megacephala* larvae powder

The histopathological results are shown in Fig. 4. Normal histological architectures were observed in the tissue sections in NC group. However, the liver tissue sections in model group showed that the number of double nuclei was increased, the hepatic cords were disarranged, liver cells expanded widely, and infiltration of large numbers of inflammatory cells was observed. Compared to NC group, kidney histopathology in model group showed that the glomeruli became atrophic or even disappeared, the number of epithelial cells was reduced, the renal proximal tubules were dilated. Histologically, the heart tissue was seen abnormally structured in model group, including cavitation and deformation in some myocardial cells, nuclear pyknosis and inflammatory cell infiltration. In model group, the brain tissue was also seen abnormally structured, including nuclear pyknosis and incomplete dissolution of nerve fibers. The caecal lesions including atrophy and breakage of the villus, irregular cell arrangement and thinning of the intestinal wall were observed in model group. SCML or VE treatment significantly attenuated these abnormal histological changes of the tissues induced by D-gal.

### Effects of SCML on oxidative stress gene expression in mice

**Fig. 5:**
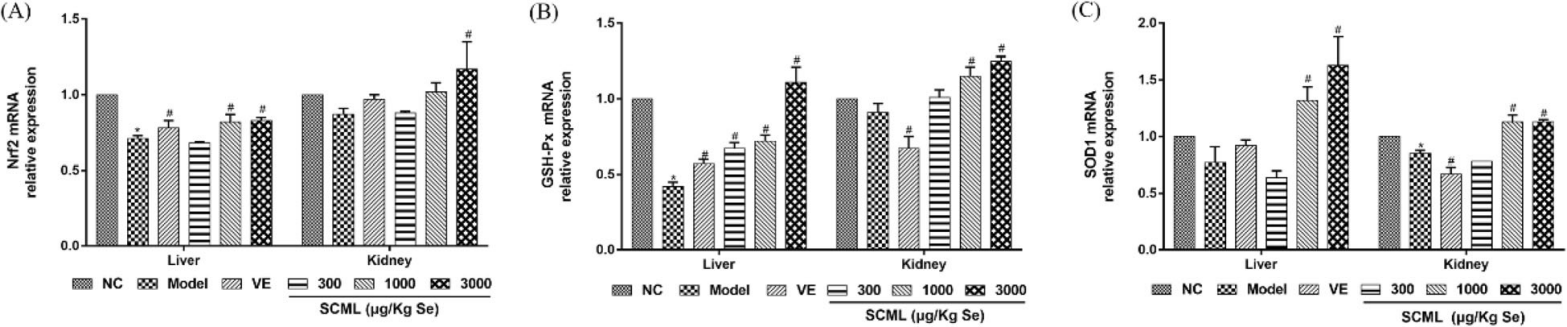
The effect of SCML on the expression of Nrf2, SOD1 and GSH-Px mRNA in the liver and kidney of the mice. **a** Nrf2 mRNA relative expression in the liver and kidney, **b** GSH-Px mRNA relative expression in the liver and kidney, **c** SOD1 mRNA relative expression in the liver and kidney. Values represent means ± SD from three independent replicates and evaluated by one-way ANOVA followed by the Duncan’s multiple-range tests. Compared with NC, ^*^P < 0.05; Compared with Model, ^#^*P* < 0.05. SCML: Selenium-enriched *C. megacephala* larvae powder

The Nrf2 pathway maintains the redox homeostasis, exerts antioxidant activity by regulating its multiple downstream cytoprotective genes, thereby plays a vital role in cell survival. The effect of SCML on oxidative stress gene expression is shown in Fig. 5. As shown in Fig. 5a, the Nrf2 expression in model group in liver was lower than that of NC group (*P* < 0.05). Except for L-SCML (300 μg/Kg Se) group in the liver, the Nrf2 expression in liver was increased all other drug treatment groups compared with model group (*P* < 0.05). The Nrf2 expression was increased in the kidney of in H-SCML (3000 μg/Kg Se) group compared to model group (*P* < 0.05). As shown in Fig. 5b, the expression of GSH-Px mRNA in the liver of model group was decreased (*P* < 0.05). After SCML treatment, the GSH-Px mRNA expression in the liver was significantly increased compare to model group (*P* < 0.05), and M-SCML (1000 μg/Kg) group and H-SCML (3000 μg/Kg Se) group in kidney was increased significantly compared to model group (*P* < 0.05). As shown in Fig. 5c, the expression of SOD1 mRNA was decreased in model group, especially in the kidney compared to NC group (*P* < 0.05). However, the expression was obviously increased in the liver of H-SCML group (3000 μg/Kg Se) and M-SCML group (1000 μg/Kg Se) compared to model group (*P* < 0.05). Significant change was also observed in SOD1 mRNA expression in the kidney of H-SCML group (3000 μg/Kg Se) and M-SCML (1000 μg/Kg Se) group compared to model group (*P* < 0.05).

### Effects of SCML on oxidative stress protein expression in mice

**Fig. 6:**
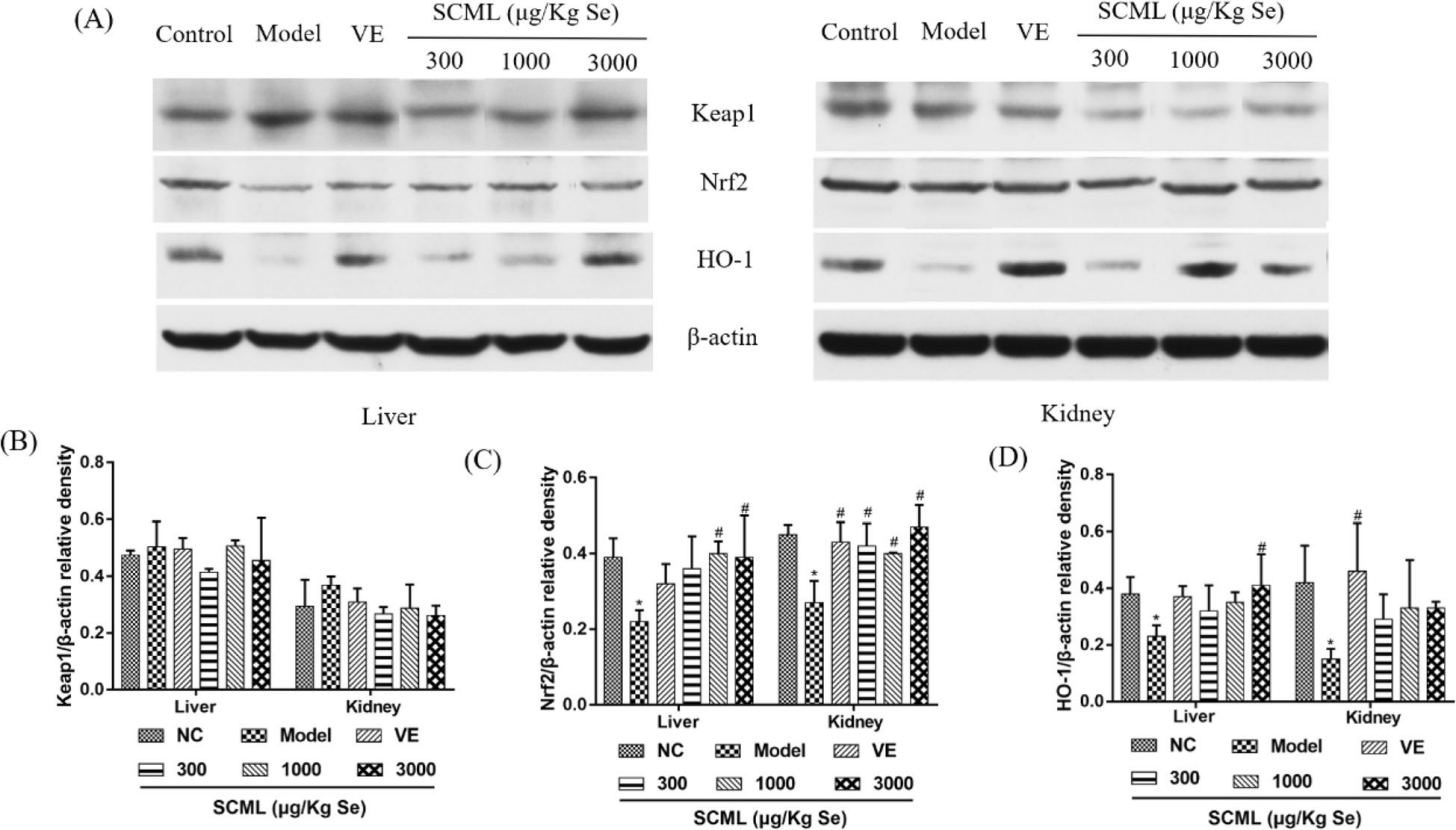
The effect of SCML on the protein expression of Keap1, Nrf2 and HO-1 in the liver and kidney tissue of the mice. **a** Protein strip, **b** Keap1/β-actin relative density, **c** Nrf2/β-actin relative density, **d** HO-1/β-actin relative density. Values represent means ± SD from three independent replicates and evaluated by one-way ANOVA followed by the Duncan’s multiple-range tests. Compared with NC, ^*^P < 0.05; Compared with Model, ^#^*P* < 0.05. SCML: Selenium-enriched *C. megacephala* larvae powder

To determine whether Nrf2 activation played a role in SCML protection against D-gal induced oxidative stress, the expression of Keap-1, Nrf2 and HO-1 in the mouse liver and kidney was detected. As shown in Fig. 6, compared with NC group, the western blot results showed that the Nrf2 and HO-1 protein expression in model group was significantly decreased (*P* < 0.05), while the Keap1 protein expression was increased in model group. After SCML or VE treatment, the Keap1 expression in the treatment groups was decreased, though the difference was not statistically significant. Compared with model group, the Nrf2 expression in the treatment groups was increased significantly (*P* < 0.05), except for L-SCML (300 μg/Kg Se) group in liver. Compared with model group, the HO-1 expression in SCML groups was increased, especially in the liver of H-SCML (3000 μg/Kg Se) group (*P* < 0.05).

### Sequencing depth and diversity

**Fig. 7:**
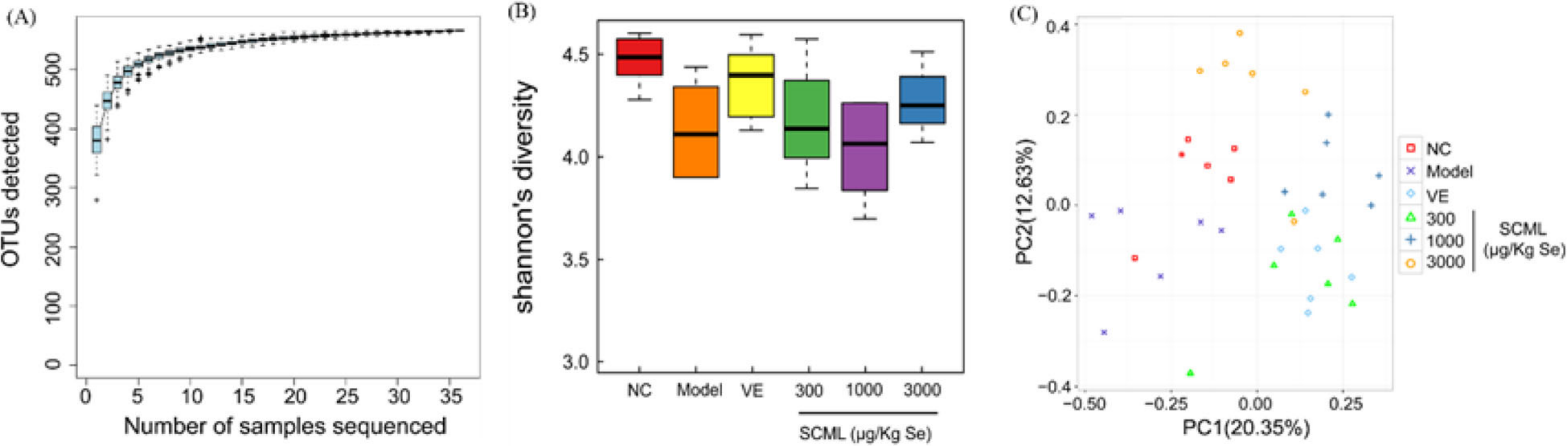
Alpha diversity of the gut microbiota and principal component analysis (PCA) plots based on abundance of operational taxonomic units (OTUs). **a** Species accumulation curves, **b** Bacterial diversity estimated by the Shannon index, **c** PCA plots. SCML: Selenium-enriched *C. megacephala* larvae powder

A total of 1,231,539 sequences from all intestinal microbiota samples were produced, averaging 34,209 sequences per sample. These sequences resulted in a mean sequence length of approximately 250 bp. Based on the Clean Tags, the cluster analysis was processed by USEARCH (v7.0.1090). The sequences were delineated into 566 operational taxonomic units (OTUs) at 97% similarity. The value of coverage for the observed OTUs was above 99.7%. The species accumulation curves showed clear asymptotes, and the curve tended to be flat or reached the plateau stage (Fig. 7a), indicating a near-complete sampling of intestinal microbial communities of mice. The boxplot of Shannon index showed that the diversity of the intestinal microbiota was decreased in model group compared to NC group, and the diversity of VE group and H-SCML (3000 μg/Kg Se) group was increased compared to model group (Fig. 7b). As shown in Fig. 7c, the contribution value of PC1 and PC2 for the sample difference was 20.35 and 12.63% respectively. All intestinal microbiota samples were presented as three distinct groups. These findings indicate that the main components of the intestinal microbiota in model group were different from those in NC group. After VE and SCML treatment, the components of the intestinal microbiota were different from those in model group, while there was an insignificant difference between H-SCML (3000 μg/Kg Se) group and NC group.

### Effects of SCML on species structures in mice

**Fig. 8:**
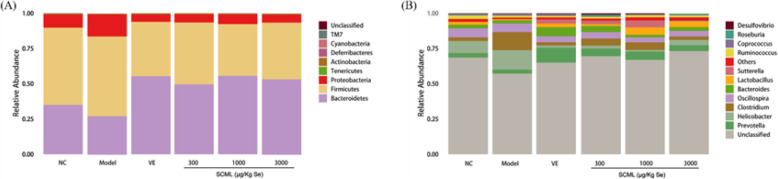
Taxonomic composition of the gut microbiome in the mice. **a** Phylum-level, **b** Species-level. SCML: Selenium-enriched *C. megacephala* larvae powder

The species profiling histogram was obtained to know the community structural composition of different groups at phylum and genus levels (Fig. 8). As shown in Fig. 8a, the most prevalent phyla in all samples were *Firmicutes* (36.67–56.65%), *Bacteroidetes (*27.20–55.64%) and *Proteobacteria* (5.39–15.69%). There were other phyla level bacteria with low abundance in the intestinal tract of mice. As shown in Fig. 8b, 11 species were used to describe the relative abundance of the intestinal microbiota at the genus level, showing that *Prevotella* (2.77–10.53%), *Helicobacter* (1.22–13.86%) and *Clostridium* (2.36–12.81%) were the most abundant, followed by *Oscillospira* (3.60–6.41%), *Bacteroides (*1.82–6.53%) and *Lactobacillus* (0.76–5.18%).

### Effects of SCML on intestinal bacteria of different classification levels in mice

As shown in Table 3, *Proteobacteria* were increased significantly at the phylum level in model group compared to NC group (*P* < 0.05). *Proteobacteria* were decreased significantly and *Bacteroidetes* were increased significantly in VE and SCML groups compared to model group (*P* < 0.05). In addition, VE group, M-SCML (1000 μg/Kg Se) group and H-SCML (3000 μg/Kg Se) group showed significant differences in *Firmicutes* compared to model group (*P* < 0.05), and M-SCML (1000 μg/Kg Se) group showed a significant difference in *Actinobacteria* compared to model group (*P* < 0.05).

**Table 3:** One-way ANOVA test of species differences at the phylum and species level (%)

	Bacteria	NC	Model	VE	SCML (μg/Kg Se)		
					300	1000	3000
Phylum	*Bacteroidetes*	35.12 ± 4.18	27.51 ± 5.75	55.42 ± 3.63^#^	49.77 ± 7.66^#^	55.66 ± 5.00^#^	53.28 ± 4.09^#^
	*Proteobacteria*	9.35 ± 1.76	15.53 ± 2.94^*^	5.32 ± 1.44^#^	5.82 ± 1.79^#^	6.89 ± 1.71^#^	5.77 ± 0.85^#^
	*Firmicutes*	54.78 ± 3.87	56.50 ± 3.84^*^	38.59 ± 3.21^#^	43.81 ± 8.03	36.67 ± 5.26^#^	40.36 ± 3.58^#^
	*Actinobacteria*	0.16 ± 0.05	0.05 ± 0.01	0.17 ± 0.06	0.27 ± 0.16	0.29 ± 0.08^#^	0.20 ± 0.04
	*Tenericutes*	0.31 ± 0.06	0.23 ± 0.07	0.39 ± 0.18	0.22 ± 0.04	0.20 ± 0.05	0.29 ± 0.06
Genus	*Bacteroides*	2.46 ± 0.28	2.36 ± 0.58	6.57 ± 1.11^#^	4.29 ± 0.49^#^	1.83 ± 0.27	2.74 ± 0.55
	*Clostridium*	2.51 ± 0.88	12.86 ± 1.98^*^	2.33 ± 1.12^#^	5.04 ± 0.95^#^	5.37 ± 3.26^#^	2.52 ± 0.84^#^
	*Helicobacter*	8.67 ± 1.77	13.70 ± 2.75^*^	1.54 ± 0.63^#^	2.05 ± 0.60^#^	1.22 ± 0.36^#^	3.73 ± 1.19^#^
	*Lactobacillus*	2.05 ± 0.62	0.75 ± 0.23	2.44 ± 0.54	1.33 ± 0.36	5.16 ± 1.20^#^	4.07 ± 1.11^#^
	*Oscillospira*	6.40 ± 0.30	6.06 ± 0.34	4.19 ± 0.44^#^	4.51 ± 0.89	3.60 ± 0.95^#^	3.93 ± 0.64^#^
	*Prevotella*	3.15 ± 0.61	2.83 ± 1.02	10.65 ± 2.71^#^	5.53 ± 1.35	5.88 ± 1.40	4.23 ± 1.26
	*Ruminococcus*	2.46 ± 0.39	1.33 ± 0.18^*^	1.09 ± 0.24	1.25 ± 0.30	1.46 ± 0.44	1.38 ± 0.21
	*Sutterella*	0.11 ± 0.04	0.08 ± 0.05	2.93 ± 1.36	2.42 ± 1.53	4.95 ± 1.86^#^	1.08 ± 0.50

Values represent means ± SE (*n* = 6) and evaluated by one-way ANOVA followed by the Duncan’s multiple-range tests. Compared with NC, ^*^P < 0.05; Compared with Model, ^#^*P* < 0.05. *SCML* Selenium-enriched *C. megacephala* larvae powder

As shown in Table 3, there was not significant alteration in *Bacteroides*, *Lactobacillus*, *Oscillospira*, *Prevotella*, and *Sutterella* at the genus level in model group compared to NC group. Compared with NC group, *Helicobacter* and *Clostridium* were increased significantly and *Ruminococcus* was decreased significantly in model group (*P* < 0.05). *Clostridium*, *Helicobacter*, and *Oscillospira* were decreased significantly in VE and SCML groups compared to model group (*P* < 0.05), while VE group and L-SCML (300 μg/Kg Se) group showed a significant difference in *Bacteroides* (*P* < 0.05). In addition, *Lactobacillus* in M-SCML (1000 μg/Kg Se) group and H-SCML (3000 μg/Kg Se) group, *Prevotella* in VE group, and *Sutterella* in M-SCML (1000 μg/Kg Se) group were all increased significantly compared to model group (*P* < 0.05). There were not significant alterations in *Ruminococcus* in VE and all SCML groups compared to model group.

### Correlation analysis of changes in flora abundance and serum biochemical indexes

In order to explain the relationship between the intestinal flora abundance changes of mice and serum biochemical indexes, Spearman correlation analysis was performed to analyze correlation between serum biochemical indexes and *Clostridium*, and *Helicobacter*, the abundance of which were significant difference in each group. The change of *Clostridium* abundance was found to be negatively correlated with GSH-Px and SOD, and positively correlated with MDA. There was not significant correlation in *Helicobacter* and serum biochemical indexes. The specific correlation analysis results were shown in the Table 4.

**Table 4:** Correlation analysis of changes in flora abundance and serum biochemical indexes

	*Clostridium*		*Helicobacter*	
	ρ	*p*	ρ	*p*
GSH-Px	−0.342	0.041	−0.238	0.162
SOD	−0.449	0.006	−0.082	0.636
MDA	0.582	< 0.001	0.161	0.349

ρ: Correlation, *p*: significance

## Discussion

The results of the present study showed that the daily behaviors of the mice in model group were different from those of the mice in NC group. In addition, the tissues of the modeled mice underwent significant pathological changes. The antioxidant system parameters including GSH-Px, SOD, T-AOC and GSH in the organ tissues or serum were decreased, while the MDA and carbonylated protein levels were increased. All these results indicated that the D-gal-induced oxidation mouse model was successfully established in the present study. VE, the monomer of which is often used as the positive control for the studies of aging in mice induced by D-gal [26, 27]. Studies showed that mice subcutaneously injected with D-gal in the neck exhibited a significant body weight declined [28]. In this study, D-gal was found to significantly inhibit weight gain in mice, however, SCML and VE could increase the body mass in varying degrees, indicating that SCML and VE could effectively enhance the constitution of aging mice. Oxidative damage appears in body organs to a large extent. Our results showed that D-gal injection for 6 weeks for mice resulted in severe histopathological changes in the organ tissues. However, SCML and VE could alleviate these D-gal-induced pathological damages in organ tissues of mice. Recent research work has demonstrated that senescent cells accumulated in various tissues of age and disease [29]. Cellular senescence is associated with age-related phenotypes causally, and decreasing senescent cells can retard tissue dysfunction and extend healthspan [30]. The results suggested SCML can delay aging by inhibiting senescent cells.

Oxidative stress is one of the major factors to contribute to cellular senescence, and a typical feature of senescence is a shift to a prooxidant redox state. The main functions of the endogenous antioxidant enzyme system are to maintain the steady state of ROS in the internal environment and remove excess ROS [31]. Therefore, numerous researches have shown that treatment with antioxidants could delay the cellular senescence [32]. Zeng et al. [33] reported that Se-enriched rice could enhance the activity of antioxidant enzymes and prevent the formation of oxidative products. In this work, the MDA and protein carbonyl levels of the mice treated with SCML were decreased, indicating that SCML could inhibit products of lipid and protein peroxidation, and stabilize the cell membrane structure. Meanwhile, activities of GSH-Px, SOD and levels of T-AOC, GSH were increased, which might mean SCML could improve the ability of enzymatic system to resist free radical via activating the antioxidant signaling pathway.

Nrf2 is a crucial molecule that mediates the response of the antioxidant system. Central to our understanding of such regulation is the activation of Nrf2 and its interaction with Keap1. Nrf2 is a transcription factor that controls the basal and inducible expression of an array of antioxidant including the HO-1, that a known target of Nrf2-regulated transcription and an antioxidative, cytoprotective protein in different types of cells, plays an important role in cytoprotection when oxidative stress occurs, and Nrf2 directly regulates its expression [34]. Recent studies have demonstrated that activation of the Nrf2 signaling pathway can protect the organism against oxidative damage [35–37]. Xu et al [38] reported that biogenic Se nanoparticles (SeNPs) synthesized by *Lactobacillus casei* ATCC 393 could protect the intestinal epithelial barrier function against oxidative damage by alleviating ROS-mediated mitochondrial dysfunction via the Nrf2 signaling pathway. It was found in our study that the gene transcriptional level of Nrf2 mRNA, the expression level of Nrf2 protein and the expression level of the antioxidant enzymes were all decreased in model group, while the expression level of Keap1 was increased, suggesting that the Nrf2 signaling pathway might be inhibited after D-gal injection under the oxidative stress condition. In addition, the expressions of Nrf2 and antioxidant enzymes were increased and the expression of Keap1 was decreased in the mice co-treated with SCML, indicating that oxidative stress was attenuated after SCML treatment. These results suggest that SCML might protect the organism against oxidative damage via the Nrf2 signaling pathway, which is consistent with the previous study by Xing et al [39]. However, the molecular signaling pathway responsible for the expression of Nrf2 was not determined and is worthy of further investigation.

Gut microbes do not age per se, but the incidences of comorbidities associated with gut microbiota tend to increase as the host grows older [40, 41]; even though it remains unclear whether microbiota alterations are cause or consequence of host aging. A multitude of researches has shown that that gut microbiota homeostasis is crucial for healthy aging and hence restoration of this homeostasis might be supportive for human longevity [42, 43]. Based on the 16S rRNA sequencing, we found that *Proteobacteria*, *Bacteroidetes* and *Firmicutes* were the dominant microorganisms in the intestinal tract of the mice at the phylum level. There are several potentially pathogenic bacteria in *Proteobacteria*, the overgrowth of which is bad for health [44]. SCML caused a significant decrease in *Proteobacteria* compared to model group, suggesting that SCML has an antagonistic effect on some pathogenic bacteria of *Proteobacteria*. Although there was not significant difference in *Bacteroidetes* and *Firmicutes* between the NC and model groups, the *Bacteroidetes* to *Firmicutes* ratio was decreased in model group. The reduction in *Bacteroidetes* and the increase in *Firmicutes* may be related to the increased ability to harvest energy from food and cause low-grade systemic inflammation, eventually damaging the organism [45, 46]. Additionally, we found that the ratio of *Bacteroidetes* to *Firmicutes* was increased in SCML groups, suggesting that intestinal flora imbalance was improved after SCML treatment.

The major bacterial genera were *Prevotella*, *Helicobacter*, *Clostridium*, *Oscillospira*, *Bacteroides*, *Lactobacillus*, *Ruminococcus* and *Sutterella*. *Helicobacter* infection aggravates dysbiosis of gut microbiome [47]. It was found in our study that in model group *Helicobacter* was increased significantly after D-gal injection compared to NC group. Meanwhile, SCML treatment reduced *Helicobacter,* which is consistent with the finding of Laure et al. [48]. Previous research showed that there were fewer *Clostridium* is known as a harmful bacterium to health [49, 50]. It was found in this work that *Clostridium* increased significantly in model group compared to that in NC group. Meanwhile, treatment with SCML could reduce *Clostridium*. Spearman correlation analysis suggested that *Clostridium* may directly or indirectly affect oxidative stress level through serum biochemical indexes. Through the analysis of data, the abundance of *Lactobacillus* in SCML treatment groups were significantly increased compared with model group. There is a large volume of published studies describing the role of *Lactobacillus*, which is a beneficial bacteria that can prevent the invasion and colonization of pathogenic bacteria and improve intestinal microbiota resulting in delayed aging [51, 52]. These results indicated that SCML could prevent imbalance in gut microbiota by promoting the growth and reproduction of probiotics and inhibiting the growth of harmful bacteria, which could reduce the oxidation damage caused by D-gal. Some research reported similar results [53–55]. In addition, SCML could increase the number of neutral bacteria such as *Bacteroides*, which prevented toxins from entering the blood by increasing intestinal wall thickness to reduce oxidative stress. However, relationship between intestinal flora and SCML requires further study.

## Conclusions

SCML could protect organism from oxidative damage. The antioxidant effect of SCML might be that SCML activated the expression of antioxidant enzymes, such as GSH-Px, HO-1, via the Nrf2 pathway. At the same time, SCML could reduce the content of lipid and protein peroxide formed by peroxidation. In addition, SCML also could improve the diversity and structure of the intestinal flora. Based on the study, SCML has the potential to be developed as a new prospect for antioxidant.

## Supplementary information

**Additional file 1.**

## Data Availability

The datasets used and/or analyzed during the current study available from the corresponding author on reasonable request.

## Abbreviations

SCML: Selenium-enriched *Chrysomyia Mgacephala* (Fabricius) Larvae powder
Se: Selenium
D-gal: D-galactose
NC: Normal control
VE: Vitamin E
MDA: Malondialdehyde
SOD: Superoxide dismutase
GSH-Px: Glutathione peroxidase
T-AOC: Total antioxidant capacity
GSH: Glutathione
Keap1: Kelch-like ECH-associated protein
Nrf2: Nuclear erythroid 2-related factor
HO-1: Hemeoxygenase-1
ICP: Inductively Coupled Plasma
qRT-PCR: Real-time quantitative PCR
one- way ANOVA: One-way analysis of variance
OUTs: Operational taxonomic units
PCA: Principal component analysis
