# Supplementary material for: Antioxidant activity of selenium-enriched Chrysomyia megacephala (Fabricius) larvae powder and its impact on intestinal microflora in D-galactose induced aging mice
Source: BMC Complement Med Ther. 2020 Aug 27;20:264. doi: 10.1186/s12906-020-03058-4 (PMC7453719; doi:10.1186/s12906-020-03058-4)
Supplement: Supplementary file 1 — Additional file 1. [file 12906_2020_3058_MOESM1_ESM.docx]

Dear Editor,

Thank you for the comments of the manuscript entitled “Antioxidant Activity of Selenium-enriched *Chrysomyia megacephala* (Fabricius) Larvae Powder and Its Impact on Intestinal Microflora in D-Galactose Induced Aging Mice” (ID: BCAM-D-20-00377R2). Those comments are very valuable and helpful for improving our paper, as well as the important guiding significance to our researches. We have made a carefully revised manuscript.

I would like to re-submit this revised manuscript, and hope it is acceptable for publication in the journal.

Looking forward to hearing from you soon.

Yours Sincerely,

Liqin Jiang

Reviewer reports:

**Reviewer 1:**

1. Line 82-83, states previous study but not cited the reference. I would suggest the author to see the guidelines of the journal how to cite the unpublished data/paper.

**The authors’ answer:** Thank you for your suggestion. The previous study about acute toxicity was published in July, so we have made relevant references. The details are as follows:

Our previous work showed that SCML was an effective organic Se source with low toxicity and high Se content[24].

24. Xie DD, Jiang LQ, Liu ZW. Study acute toxic and antioxidant activity effect of selenium enrichment calliphoridae powder on mice. Studies of Trace Elements and Health. 2020; 37(04):1-3.

2. Line 160-161, change the sentence "The results were analyzed by using the 2-ΔΔCT assay" to "The results were calculated by using the 2-ΔΔCT method."

**The authors’ answer:** Thank you for your suggestion. We have changed the sentence "The results were analyzed by using the 2^-ΔΔCT^ assay" to "The results were calculated by using the 2^-ΔΔCT^ method " in line 161-162.

**Reviewer 3:**

23. Line 133 correct conclucted

The authors' answer: Thank you for your kindness, we have corrected "conclucted" to "conculcated" in line 128.

Change "conculcated" to "conducted"

**The authors’ answer:** Thank you for your suggestion. We have changed "conculcated" to "conducted" in line 128. The details are as follows:

The experiments were conducted at 9:00-12:00. A certain amount of SCML and gellan gum were weighed precisely and dissolved in purified water, heated slightly to a suspension.

28. Line 165 one step of PCR is missing

The authors' answer: Thank you for your valuable advice. We have added the step of PCR in line 154 and 155. The details are as follows:
RNA was reverse transcribed into cDNA using RevertAid First Strand cDNA Synthesis Kit.

each PCR cycle has 3 steps: denaturation, annealing and extension but only 2 steps were given

**The authors’ answer:** Thank you for your suggestion, we have added the step of PCR in line 159. The details are as follows:

Total RNA was extracted from the liver and kidney tissues using Trizol reagent. RNA was reverse transcribed into cDNA using RevertAid First Strand cDNA Synthesis Kit. Real-time quantitative PCR (qRT-PCR) was performed using FastStart Universal SYBR Green Master (Rox) and the ABI7900Faxt Sequence Detection system. The thermal cycle condition was one cycle at 95℃ for 10 min, followed by 40 cycles of amplification at 95℃ for 15s, and then 60℃ for 30s. And the dissolution curve started from 60°C, then ascending to 95°C at 0.3°C/15 s. All samples were run in triplicate in each experiment. Values were normalized to that for GAPDH. The sequences of the primers used are shown in Table 2. The results were calculated by using the 2^-ΔΔCT^ method.

50. Line 339 Replace wasn't with "was no"

The authors' answer: Thank you for your kindness, we have replaced "wasn't" with "was no" in line 329.

=> change "was no" to "was not"

**The authors’ answer:** Thank you for your kindness, we have change "was no" to "was not" in line 330. The details are as follows:

There was not significant correlation in *Helicobacter* and serum biochemical indexes.

52. Line347-349 Rewrite this sentence to clearly bring out its purpose. It appears incomplete

The authors' answer: Thank you for your kind suggestion. We have rewritten the sentence in line 339-343. For your earliest convenience, you could also read it here as below:

Studies showed that mice subcutaneously injected the D-gal neck exhibited a significant body weight declined[27]. In the study, D-gal was found to significantly inhibit weight gain in mice, however, SCML and VE could increase the body mass in varying degrees, indicating that SCML and VE could effectively enhance the constitution of aging mice.

27. Liu J, Chen D, Wang ZK, Chen CY, Ning DL, Zhao SL. Protective effect of walnut on d-galactose-induced aging mouse model. Food Sci. Nutr. 2019;7(3):969-976.
=> Correct these sentences Change "injected the D-gal neck exhibited" to " injected with D-gal in the neck exhibited" Change "In the study" to "in this study"

**The authors’ answer:** Thank you for your suggestion, we have changed "injected the D-gal neck exhibited" to " injected with D-gal in the neck exhibited" and changed "In the study" to "in this study" in line 341 and 342. The details are as follows:

Studies showed that mice subcutaneously injected with D-gal in the neck exhibited a significant body weight declined[28]. In this study, D-gal was found to significantly inhibit weight gain in mice, however, SCML and VE could increase the body mass in varying degrees, indicating that SCML and VE could effectively enhance the constitution of aging mice.

28. Liu J, Chen D, Wang ZK, Chen CY, Ning DL, Zhao SL. Protective effect of walnut on d-galactose-induced aging mouse model. Food Sci. Nutr. 2019;7(3):969-976.
